# New Appliances and Things Medical

**Published:** 1903-05-30

**Authors:** 


### NEW APPLIANCES AND THINGS MEDICAL.

**[We shall be glad to receive at our Office, 28 & 29 Southampton Street, Strand, London, W.C., from the manufacturers, specimens of all new preparations and appliances which may be brought out from time to time.]**

### MUNICH LAGER BEERS.

(The Munich Lionbrew Company, 62 Holborn Viaduct, London, E.C.)

We have examined samples of the above beers and find that they fulfill the conditions which are required of a safe and agreeable beverage for ordinary use. These beers are brewed under thoroughly hygienic conditions and on the most approved principles. The "sedimentary" or "bottom-yeast" method of fermentation which is employed by the Lionbrew Company has distinct advantages over the surface fermentation method usually employed in this country. By the former method abnormal or pathogenic fermentation owing to the activity of impure yeasts is less likely to occur, the flavour of the beer is improved, and the products of fermentation little calculated to disagree. For consumption by city dwellers there can be no question that these light German beers are more suitable than most of our heavy English beers, which contain a higher percentage of alcohol and a larger proportion of maltose and dextrine. It is these carbohydrates which possess the fattening properties of beers and which give the habitual beer-drinker the characteristic bloated and puffy appearance. The smaller the proportion, therefore, of unconverted carbohydrates and the lower the percentage of alcohol, the safer for consumption is the beer. The beers brewed by the Lionbrew Company are very fine samples of German lager beers. We have seldom tasted a pleasanter beer even in Germany itself under local conditions and fresh from the cask. We recommend them especially for those whose sedentary occupations do not permit of our stronger English varieties.

